# Correction to: Investigating the parameter space of evolutionary algorithms

**DOI:** 10.1186/s13040-019-0210-3

**Published:** 2019-11-18

**Authors:** Moshe Sipper, Weixuan Fu, Karuna Ahuja, Jason H. Moore

**Affiliations:** 1 Institute for Biomedical Informatics, University of Pennsylvania, Philadelphia, PA 19104-6021 USA; 2 Department of Computer Science, Ben-Gurion University, 8410501 Beer Sheva, Israel

**Correction to: BioData Mining (2018) 11:2.**

**DOI 10.1186/s13040-018-0164-x**

Following publication of the original article [1], an error was reported in one of the experiments.

On page 11 of [1] we described the following experiment:

DEAP: For each of the 110 good parameter sets found, generate *pop_size* × *gen_count* × [1, 5] random solutions and check how many of them pass the same 5-problem criterion employed above.

We showed that no random tree thus created met the success criterion described in the paper. However, it seems that we inadvertently used DEAP defaults, one of which is a maximal tree height of 2. This produces small, shallow trees, and it can be argued that they stand little chance of succeeding in solving the problems addressed in the paper.

We reran said experiment, this time running eight sub-experiments:

## Generate random trees through grow method

- with minimal height 1, maximal height 5,
- with minimal height 1, maximal height 10,
- with minimal height 2, maximal height 5,
- with minimal height 2, maximal height 10.

## Generate random trees through full method

- with minimal height 1, maximal height 5,
- with minimal height 1, maximal height 10,
- with minimal height 2, maximal height 5,
- with minimal height 2, maximal height 10.

Despite the much larger trees thus created our results have been confirmed, namely, none of the random trees met the success criterion.
